# Adaptive evolution of bat dipeptidyl peptidase 4 (dpp4): implications for the origin and emergence of Middle East respiratory syndrome coronavirus

**DOI:** 10.1186/1743-422X-10-304

**Published:** 2013-10-10

**Authors:** Jie Cui, John-Sebastian Eden, Edward C Holmes, Lin-Fa Wang

**Affiliations:** 1 Marie Bashir Institute for Infectious Diseases and Biosecurity, School of Biological Sciences and Sydney Medical School, The University of Sydney, Sydney, NSW 2006, Australia; 2 Duke-NUS Graduate Medical School, Singapore 169857, Singapore; 3 CSIRO Livestock Industries, Australian Animal Health Laboratory, Geelong, VIC 3220, Australia

**Keywords:** MERS-CoV, Bats, Arms-race, Adaptive evolution, Emergence

## Abstract

**Background:**

The newly emerged Middle East respiratory syndrome coronavirus (MERS-CoV) that first appeared in Saudi Arabia during the summer of 2012 has to date (20th September 2013) caused 58 human deaths. MERS-CoV utilizes the dipeptidyl peptidase 4 (DPP4) host cell receptor, and analysis of the long-term interaction between virus and receptor provides key information on the evolutionary events that lead to the viral emergence.

**Findings:**

We show that bat *DPP4* genes have been subject to significant adaptive evolution, suggestive of a long-term arms-race between bats and MERS related CoVs. In particular, we identify three positively selected residues in DPP4 that directly interact with the viral surface glycoprotein.

**Conclusions:**

Our study suggests that the evolutionary lineage leading to MERS-CoV may have circulated in bats for a substantial time period.

## Main text

Middle East respiratory syndrome coronavirus (MERS-CoV) [1], first described by the World Health Organization (WHO) on 23rd September 2012 [2,3], has to date (20th September 2013) caused 130 laboratory-confirmed human infections with 58 deaths (http://www.who.int/csr/don/2013_09_20/en/index.html). MERS-CoV belongs to lineage C of the genus *Betacoronavirus* in the family *Coronaviridae*, and is closely related to *Tylonycteris* bat coronavirus HKU4 (BtCoV-HKU4), *Pipistrellus* bat coronavirus HKU5 (Bt-HKU5) [4,5] and CoVs in *Nycteris* bats [6], suggestive of a bat-origin [6]. Unlike severe acute respiratory syndrome (SARS) CoV which uses the angiotensin-converting enzyme 2 (ACE2) receptor for cell entry [7], MERS-CoV employs the dipeptidyl peptidase 4 receptor (DPP4; also known as CD26), and recent work has demonstrated that expression of both human and bat DPP4 in non-susceptible cells enabled viral entry [8].

Cell-surface receptors such as DPP4 play a key role in facilitating viral invasion and tropism. As a consequence, the long-term co-evolutionary dynamics between hosts and viruses often leave evolutionary footprints in both receptor-encoding genes of hosts and the receptor-binding domains (RBDs) of viruses in the form of positively selected amino acid residues (i.e. adaptive evolution). For example, signatures of recurrent positive selection have been observed in *ACE2* genes in bats [9], supporting the past circulation of SARS related CoVs in bats. To better understand the origins of MERS-CoV, as well as their potentially long-term (compared to short-term which lacks virus-host interaction) evolutionary dynamics with bat hosts [5,10], we studied the molecular evolution of *DPP4* across the mammalian phylogeny.

**Figure 1:**
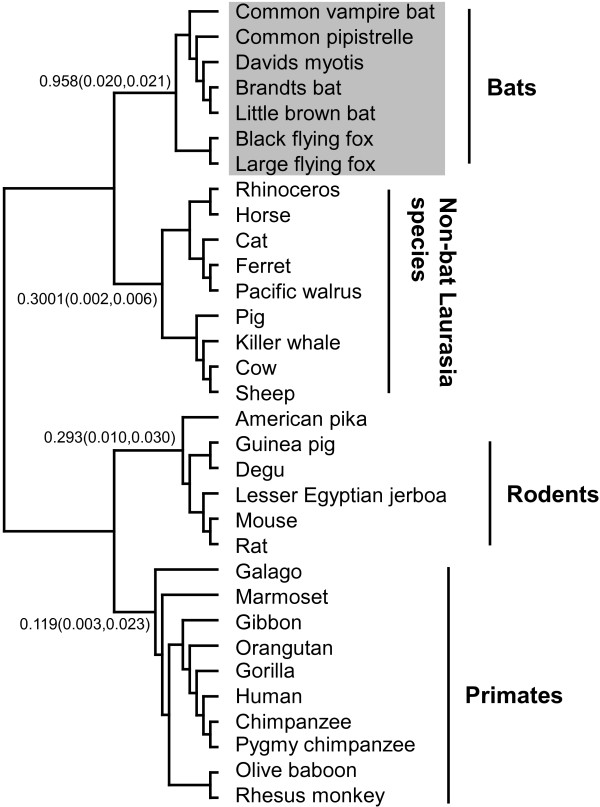
**Selection pressures on *DPP4 *during mammalian evolution.** Ratios of nonsynonymous (d_N_) to synonymous (d_S_) nucleotide substitutions per site (d_N_/d_S_) are shown on four major ancestral branches; d_N_ and d_S_ numbers are also given in parentheses. Values for individual lineages are given in Table 2. *DPP4* sequences of bat origin are shaded.

We first analyzed the selection pressures acting on bat *DPP4* genes using the ratio of nonsynonymous (d_N_) to synonymous (d_S_) nucleotide substitutions per site (ratio d_N_/d_S_), with d_N_ > d_S_ indicative of adaptive evolution. The complete *DPP4* mRNA sequence of the common pipistrelle (*Pipistrellus pipistrellus*) was downloaded from GenBank (http://www.ncbi.nlm.nih.gov/genbank/) along with that of the common vampire bat (*Desmodus rotundus*) from one transcriptome database (http://www.ncbi.nlm.nih.gov/bioproject/178123). These sequences were then used to mine and extract DPP4 mRNA transcripts from a further five bat genomes (Table 1) using tBLASTn and GeneWise [11]. The complete DPP4 genes of bats and non-bat reference genomes from a range of mammalian species (Table 1) were aligned using MUSCLE [12] guided by translated amino acid sequences (*n* = 32; 727 amino acids). We then compared a series of models within a maximum likelihood framework [13], incorporating the published mammalian species tree [14-16]. This analysis (the Free Ratio model) revealed that the d_N_/d_S_ value on the bat lineage (0.96) was four times greater than the mammalian average (Figure 1). The higher d_N_/d_S_ ratios leading to bats (Table 2) during mammalian evolution accord with the growing body of data [5,6,17,18] that the newly emerged MERS-CoV ultimately has a bat-origin.

**Table 1:** **Sequences used in the evolutionary analysis of *DDP4***

**Common name**	**Species name**	**Family**	**Accession no.**
Sheep	*Ovis aries*	Bovidae	XM_004004660
Killer whale	*Orcinus orca*	Delphinidae	XM_004283621
Cow	*Bos taurus*	Bovidae	NM_174039
Pig	*Sus scrofa*	Suidae	NM_214257
Pacific walrus	*Odobenus rosmarus divergens*	Odobenidae	XM_004410199
Ferret	*Mustela putorius furo*	Mustelidae	DQ266376
Cat	*Felis catus*	Felidae	NM_001009838
Horse	*Equus caballus*	Equidae	XM_001493999
Rhinoceros	*Ceratotherium simum*	Rhinocerotidae	XM_004428264
Large flying fox	*Pteropus vampyrus*	Pteropodidae	ENSPVAG00000002634
Black flying fox	*Pteropus alecto*	Pteropodidae	KB031068
Common vampire bat	*Desmodus rotundus*	Phyllostomidae	GABZ01004546
Brandt’s bat	*Myotis brandtii*	Vespertilionidae	KE161360
David’s myotis	*Myotis davidii*	Vespertilionidae	KB109552
Little brown bat	*Myotis lucifugus*	Vespertilionidae	GL429772
Common pipistrelle	*Pipistrellus pipistrellus*	Vespertilionidae	KC249974
Guinea pig	*Cavia porcellus*	Caviidae	XM_003478564
Degu	*Octodon degus*	Octodontidae	XM_004629976
Lesser Egyptian jerboa	*Jaculus jaculus*	Dipodidae	XM_004651712
Mouse	*Mus musculus*	Muridae	BC022183
Rat	*Rattus norvegicus*	Muridae	NM_012789
Human	*Homo sapiens*	Hominidae	NM_001935
Chimpanzee	*Pan troglodytes*	Hominidae	GABE01002695
Pygmy chimpanzee	*Pan paniscus*	Hominidae	XM_003820939
Gorilla	*Gorilla gorilla gorilla*	Hominidae	XM_004032706
Orangutan	*Pongo abelii*	Hominidae	NM_001132869
Gibbon	*Nomascus leucogenys*	Hylobatidae	XM_003266171
Olive baboon	*Papio anubis*	Cercopithecidae	XM_003907539
Rhesus monkey	*Macaca mulatta*	Cercopithecidae	JU474559
Galago	*Otolemur garnettii*	Galagidae	XM_003795172
Marmoset	Callithrix jacchus	Cebidae	XM_002749392
American pika	*Ochotona princeps*	Ochotonidae	XM_004577330

**Table 2:** **Numbers of nonsynonymous (d**_**N**_**) and synonymous (d**_**S**_**) substitutions per site *DPP4 *genes in different mammals**

**Common name**	**d**_**N**_	**d**_**S**_	**d**_**N**_**/d**_**S**_
Sheep	0.004	0.013	0.280
Killer whale	0.023	0.039	0.595
Cow	0.003	0.016	0.157
Pig	0.027	0.109	0.246
Pacific walrus	0.014	0.053	0.260
Ferret	0.015	0.064	0.235
Cat	0.021	0.081	0.258
Horse	0.016	0.055	0.290
Rhinoceros	0.017	0.044	0.385
Large flying fox	0.005	0.001	3.561
Black flying fox	0.004	0.008	0.487
Common vampire bat	0.042	0.125	0.500
Brandt’s bat	0.006	0.012	0.463
David’s myotis	0.010	0.028	0.380
Little brown bat	0.007	0.007	0.943
Common pipistrelle	0.031	0.066	0.470
Guinea pig	0.018	0.078	0.238
Degu	0.016	0.128	0.122
Lesser Egyptian jerboa	0.023	0.179	0.131
Mouse	0.019	0.093	0.206
Rat	0.027	0.110	0.248
Human	0.001	0.007	0.086
Chimpanzee	0.000	0.002	0.000
Pygmy chimpanzee	0.001	0.000	ND
Gorilla	0.003	0.004	0.863
Orangutan	0.002	0.000	ND
Gibbon	0.003	0.009	0.344
Olive baboon	0.000	0.005	0.000
Rhesus monkey	0.000	0.004	0.000
Galago	0.022	0.149	0.149
Marmoset	0.009	0.053	0.160
American pika	0.036	0.229	0.156

ND: Not determined because no synonymous substitutions are present.

**Figure 2:**
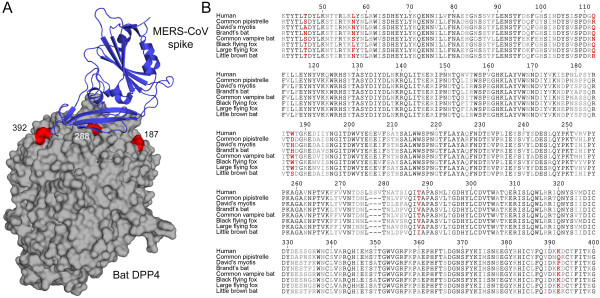
**Interaction of bat DPP4 and MERS-CoV spike protein receptor-binding domain and the location of positively selected sites.** The structure was displayed using PyMol v1.6 (http://www.pymol.org/). **(A)** Homology model showing the structural interactions between bat DPP4 (from common pipistrelle) coloured grey and MERS-CoV spike protein receptor-binding domain coloured blue. The three positively selected residues (positions 187, 288 and 392) located within the interface where the virus-host interact are highlighted as red. **(B)** Protein alignment of human DPP4 compared to that of seven bat species showing RBD spanning codons 41 – 400. Conserved and variable positions are shown in black and grey text, respectively, and residues under positive selection are coloured red.

We next analysed the selection pressures at individual amino acid sites in bat DPP4. Using the Bayesian FUBAR method [19] in HyPhy package [20], we identified six codons that were assigned d_N_/d_S_ > 1 with higher posterior probability (a strict cut-off of 95% in this analysis) (Table 3). To identify those sites under positive selection that may interact directly with MERS-CoV-like spike protein, bat DPP4 (from the common pipistrelle) was modelled against the structure of the human DPP4/MERS-CoV spike complex [21] (Figure 2A). This revealed that three of the six positive selected residues (position 187, 288 and 392) were located at the interface between bat DPP4 and MERS-CoV RBD (receptor binding domain) (Figure 2). These residues therefore provide direct evidence of a long-term co-evolutionary history between viruses and their hosts. We also observed several variable regions (Figure 2B) within the bat RBD, that may also have resulted from virally-induced selection pressure and which merit additional investigation in a larger data set.

**Table 3:** **Putatively positive selected *DPP4 *codons in bats**

**Codon position**^***a***^	**Posterior probability**^***b***^	**d**_**N**_**/d**_**S**_
46	0.97	14.95
57	0.97	13.13
112	0.94	10.27
187	0.95	8.55
288	0.98	13.90
392	0.97	14.63

^*a*^Codon position corresponding to the human DPP4 (NP_001926) protein sequence.

^*b*^Posterior probability of residues assigned a d_N_/d_S_ ratio greater than 1.

Our analysis therefore suggests that the evolutionary lineage leading to current MERS-CoV co-evolved with bat hosts for an extended time period, eventually jumping species boundaries to infect humans and perhaps through an intermediate host. As such, the emergence of MERS-CoV may parallel that of the related SARS-CoV [22]. Although one bat species, *Taphozous erforatus*, in Saudi Arabia has been found to harbour a small *RdRp* (RNA-Dependent RNA Polymerase) fragment of MERS-CoV [17], a larger viral sampling of bats and other animals with close exposure to humans, including dromedary camels were serological evidence for MERS-CoV has been identified [23], are clearly needed to better understand the viral transmission route. Alternatively, it is possible that the adaptive evolution present on the bat DPP4 was due to viruses other than MERS-CoVs, and which will need to be better assessed when a larger number of viruses are available for analysis. Overall, our study provides evidence that a long-term evolutionary arms race likely occurred between MERS related CoVs and bats.

## Competing interests

The authors declare that they have no competing interests.

## Authors’ contributions

JC and LFW designed the research. JC and JSE analysed the data. JC and ECH drafted the manuscript. All authors read and approved the final manuscript.

## References

[B1] de Groot RJ Baker SC Baric RS Brown CS Drosten C Enjuanes L Fouchier RA Galiano M Gorbalenya AE Memish ZA Perlman S Poon LL Snijder EJ Stephens GM Woo PC Zaki AM Zambon M Ziebuhr J Middle East respiratory syndrome coronavirus (MERS-CoV): announcement of the coronavirus study group J Virol 2013 10 7790 7792 10.1128/JVI.01244-13 23678167 PMC3700179

[B2] Zaki AM van Boheemen S Bestebroer TM Osterhaus AD Fouchier RA Isolation of a novel coronavirus from a man with pneumonia in Saudi Arabia N Engl J Med 2012 10 1814 1820 10.1056/NEJMoa1211721 23075143

[B3] Bermingham A Chand MA Brown CS Aarons E Tong C Langrish C Hoschler K Brown K Galiano M Myers R Pebody RG Green HK Boddington NL Gopal R Price N Newsholme W Drosten C Fouchier RA Zambon M Severe respiratory illness caused by a novel coronavirus, in a patient transferred to the United Kingdom from the Middle East, September 2012 Euro Surveill 2012 10 20290 23078800

[B4] van Boheemen S de Graaf M Lauber C Bestebroer TM Raj VS Zaki AM Osterhaus AD Haagmans BL Gorbalenya AE Snijder EJ Fouchier RA Genomic characterization of a newly discovered coronavirus associated with acute respiratory distress syndrome in humans mBio 2012 10 e00473-12 10.1128/mBio.00473-12 23170002 PMC3509437

[B5] Lau SK Li KS Tsang AK Lam CS Ahmed S Chen H Chan KH Woo PC Yuen KY Genetic characterization of *Betacoronavirus* lineage C viruses in bats reveals marked sequence divergence in the spike protein of *Pipistrellus* bat coronavirus HKU5 in Japanese Pipistrelle: implications for the origin of the novel Middle East respiratory syndrome coronavirus J Virol 2013 10 8638 8650 10.1128/JVI.01055-13 23720729 PMC3719811

[B6] Annan A Baldwin HJ Corman VM Klose SM Owusu M Nkrumah EE Badu EK Anti P Agbenyega O Meyer B Oppong S Sarkodie YA Kalko EK Lina PH Godlevska EV Reusken C Seebens A Gloza-Rausch F Vallo P Tschapka M Drosten C Drexler JF Human betacoronavirus 2c EMC/2012-related viruses in bats, Ghana and Europe Emerg Infect Dis 2013 10 456 459 10.3201/eid1903.121503 23622767 PMC3647674

[B7] Müller MA Raj VS Muth D Meyer B Kallies S Smits SL Wollny R Bestebroer TM Specht S Suliman T Zimmermann K Binger T Eckerle I Tschapka M Zaki AM Osterhaus AD Fouchier RA Haagmans BL Drosten C Human coronavirus EMC does not require the SARS-coronavirus receptor and maintains broad replicative capability in mammalian cell lines mBio 2012 10 e00515-12 10.1128/mBio.00515-12 23232719 PMC3520110

[B8] Raj VS Mou H Smits SL Dekkers DH Müller MA Dijkman R Muth D Demmers JA Zaki A Fouchier RA Thiel V Drosten C Rottier PJ Osterhaus AD Bosch BJ Haagmans BL Dipeptidyl peptidase 4 is a functional receptor for the emerging human coronavirus-EMC Nature 2013 10 251 254 10.1038/nature12005 23486063 PMC7095326

[B9] Demogines A Farzan M Sawyer SL Evidence for ACE2-utilizing coronaviruses (CoVs) related to severe acute respiratory syndrome CoV in bats J Virol 2012 10 6350 6353 10.1128/JVI.00311-12 22438550 PMC3372174

[B10] Kindler E Jónsdóttir HR Muth D Hamming OJ Hartmann R Rodriguez R Geffers R Fouchier RA Drosten C Müller MA Dijkman R Thiel V Efficient replication of the novel human betacoronavirus EMC on primary human epithelium highlights its zoonotic potential mBio 2013 10 e00611 e00612 23422412 10.1128/mBio.00611-12 PMC3573664

[B11] Birney E Clamp M Durbin R GeneWise and Genomewise Genome Res 2004 10 988 995 10.1101/gr.1865504 15123596 PMC479130

[B12] Edgar RC MUSCLE: multiple sequence alignment with high accuracy and high throughput Nucleic Acids Res 2004 10 1792 1797 10.1093/nar/gkh340 15034147 PMC390337

[B13] Yang Z PAML 4: phylogenetic analysis by maximum likelihood Mol Biol Evol 2007 10 1586 1591 10.1093/molbev/msm088 17483113

[B14] Murphy WJ Pevzner PA O'Brien SJ Mammalian phylogenomics comes of age Trends Genet 2004 10 631 639 10.1016/j.tig.2004.09.005 15522459

[B15] Teeling EC Springer MS Madsen O Bates P O'brien SJ Murphy WJ A molecular phylogeny for bats illuminates biogeography and the fossil record Science 2005 10 580 584 10.1126/science.1105113 15681385

[B16] Perelman P Johnson WE Roos C Seuánez HN Horvath JE Moreira MA Kessing B Pontius J Roelke M Rumpler Y Schneider MP Silva A O'Brien SJ Pecon-Slattery J A molecular phylogeny of living primates PLoS Genet 2011 10 e1001342 10.1371/journal.pgen.1001342 21436896 PMC3060065

[B17] Memish ZA Mishra N Olival KJ Fagbo SF Kapoor V Epstein JH AlHakeem R Al Asmari M Islam A Kapoor A Briese T Daszak P Al Rabeeah AA Lipkin WI Middle east respiratory syndrome coronavirus in bats, Saudi Arabia Emerg Infect Disin press 10.3201/eid1911.131172 PMC3837665 24206838

[B18] Ithete NL Stoffberg S Corman VM Cottontail VM Richards LR Schoeman MC Drosten C Drexler JF Preiser W Close relative of human middle East respiratory syndrome coronavirus in bat, South Africa Emerg Infect Dis 2013 10 1697 1699 10.3201/eid1910.130946 24050621 PMC3810765

[B19] Murrell B Moola S Mabona A Weighill T Sheward D Kosakovsky Pond SL Scheffler K FUBAR: a fast, unconstrained bayesian approximation for inferring selection Mol Biol Evol 2013 10 1196 1205 10.1093/molbev/mst030 23420840 PMC3670733

[B20] Pond SL Frost SD Muse SV HyPhy: hypothesis testing using phylogenies Bioinformatics 2005 10 676 679 10.1093/bioinformatics/bti079 15509596

[B21] Wang N Shi X Jiang L Zhang S Wang D Tong P Guo D Fu L Cui Y Liu X Arledge KC Chen YH Zhang L Wang X Structure of MERS-CoV spike receptor-binding domain complexed with human receptor DPP4 Cell Res 2013 10 986 993 10.1038/cr.2013.92 23835475 PMC3731569

[B22] Cui J Han N Streicker D Li G Tang X Shi Z Hu Z Zhao G Fontanet A Guan Y Wang L Jones G Field HE Daszak P Zhang S Evolutionary relationships between bat coronaviruses and their hosts Emerg Infect Dis 2007 10 1526 1532 10.3201/eid1310.070448 18258002 PMC2851503

[B23] Reusken CB Haagmans BL Müller MA Gutierrez C Godeke GJ Meyer B Muth D Raj VS Vries LS Corman VM Drexler JF Smits SL El Tahir YE De Sousa R van Beek J Nowotny N van Maanen K Hidalgo-Hermoso E Bosch BJ Rottier P Osterhaus A Gortázar-Schmidt C Drosten C Koopmans MP Middle East respiratory syndrome coronavirus neutralising serum antibodies in dromedary camels: a comparative serological study Lancet Infect Dis 2013 10 859 866 10.1016/S1473-3099(13)70164-6 23933067 PMC7106530

